# Supplementary material for: On the Use of Pulsed UV or Visible Light Activated Gas Sensing of Reducing and Oxidising Species with WO3 and WS2 Nanomaterials
Source: Sensors (Basel). 2021 May 27;21(11):3736. doi: 10.3390/s21113736 (PMC8199237; doi:10.3390/s21113736)
Supplement: Supplementary file 1 [file sensors-21-03736-s001.zip › sensors-1198561-supplementary.pdf]

On the Use of Pulsed UV or Visible Light Activated Gas Sensing of Reducing and Oxidising Species with WO_3 and WS_2 Nanomaterials

Ernesto González [1], Juan Casanova-Chafer [1], Aanchal Alagh [1], Alfonso Romero [1], Xavier Vilanova [1,*], Selene Acosta [2], Damien Cossement [3], Carla Bittencourt [2] and Eduard Llobet [1]

[1] Electronic Engineering, Uiversitat Rovira i Virgili, 43007 Tarragona, Spain; ernesto.gonzalez@urv.cat (E.G.); juan.casanova@urv.cat (J.C.-C.); alagh.aanchal@urv.cat (A.A.); alfonso.romero@urv.cat (A.R.); eduard.llobet@urv.cat (E.L.)

[2] Chimie des Interactions Plasma e Surface (ChIPS), Research Institute for Materials Science and Engineering, Université de Mons, 3, 7000 Mons, Belgium; selene.acostamorales@umons.ac.be (S.A.); carla.bittencourt@umons.ac.be (C.B.)

[3] Materia Nova, Parc Initialis, 3, 7000 Mons, Belgium; damien.cossement@materianova.be (D.C.)

* Correspondence: xavier.vilanova@urv.cat; Tel.: +34-977-558-502

Citation: González, E.; Casanova-Chafer, J.; Alagh, A.; Romero, A.; Vilanova, X.; Acosta, S.; Cossement, D.; Bittencourt, C.; Llobet, E. On the Use of Pulsed UV or Visible Light Activated Gas Sensing of Reducing and Oxidising Species with WO_3 and WS_2 Nanomaterials. *Sensors* **2021**, *21*, 3736. https://doi.org/10.3390/s21113736

Academic Editor: Giovanni Neri

Received: 10 April 2021
Accepted: 25 May 2021
Published: 27 May 2021

Publisher's Note: MDPI stays neutral with regard to jurisdictional claims in published maps and institutional affiliations.

Copyright: © 2021 by the authors. Licensee MDPI, Basel, Switzerland. This article is an open access article distributed under the terms and conditions of the Creative Commons Attribution (CC BY) license (http://creativecommons.org/licenses/by/4.0/).

Experimental setup

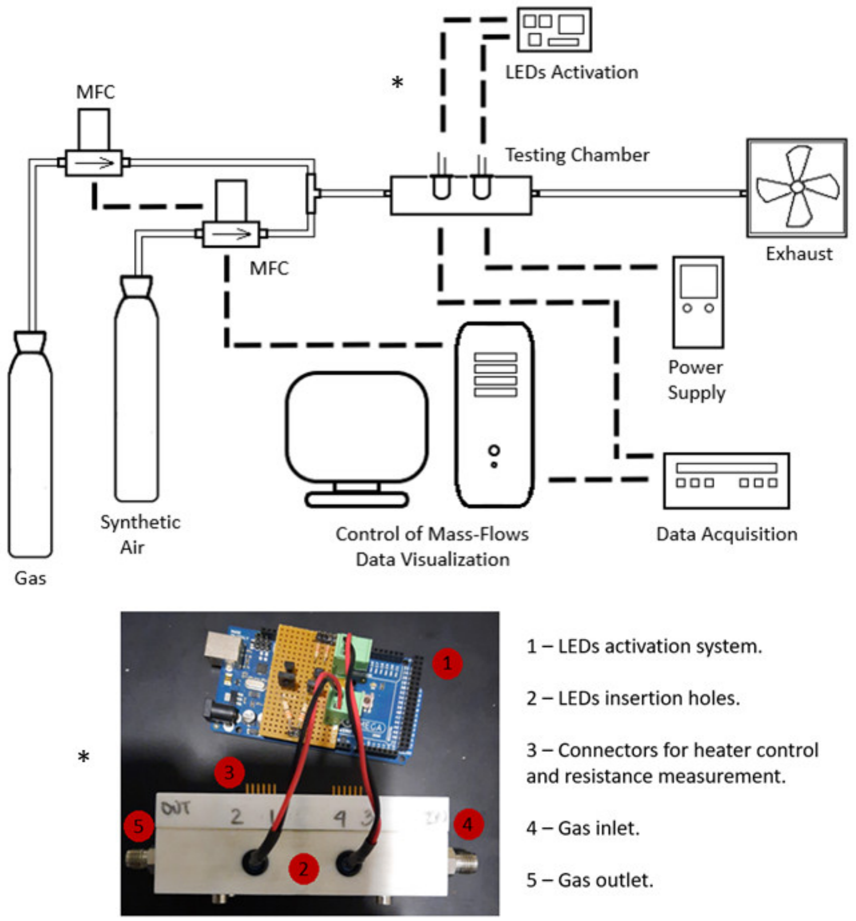

Figure 1. Gas measurement system used to perform the presented pulsed light modulated gas sensing methodology.

EDX and XRD results

EDX spectrum from Figure S2a confirms that the WO_3 pristine sensor composition is just tungsten and oxygen, being the sample free of any contaminant. In addition, diffraction peaks from the XRD spectrum in Figure S3a correspond to the monoclinic phase of the WO_3 (ICDD 43-1035).

On the other hand, the EDX spectrum shown in Figure S2b shows that the composition of the WS_2 nanoflakes consists of tungsten and sulfur, free of oxygen content. From the XRD spectrum in Figure S3b, many reflection peaks are related to the hexagonal P63/mmc space group, which confirm the presence of the 2H phase of the WS_2. Moreover, some of the peaks present in the spectrum belong to the alumina substrate. No peak in the spectrum is related to WO_3 impurities.

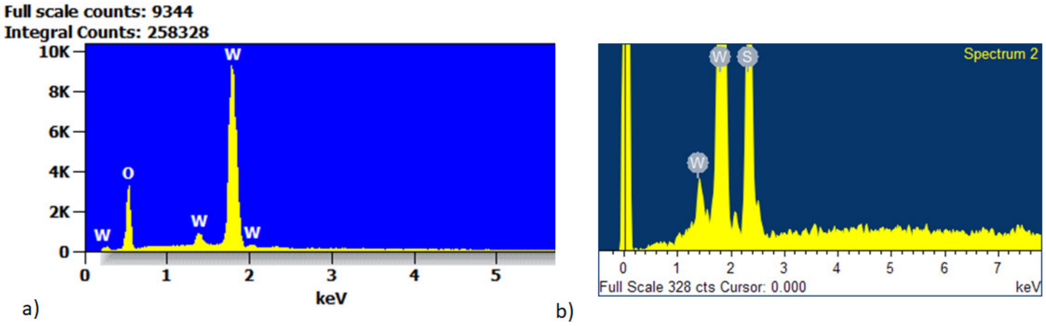

Figure 2. EDX analysis spectra from a) WO_3 pristine sensor and b) WS_2 sensor.

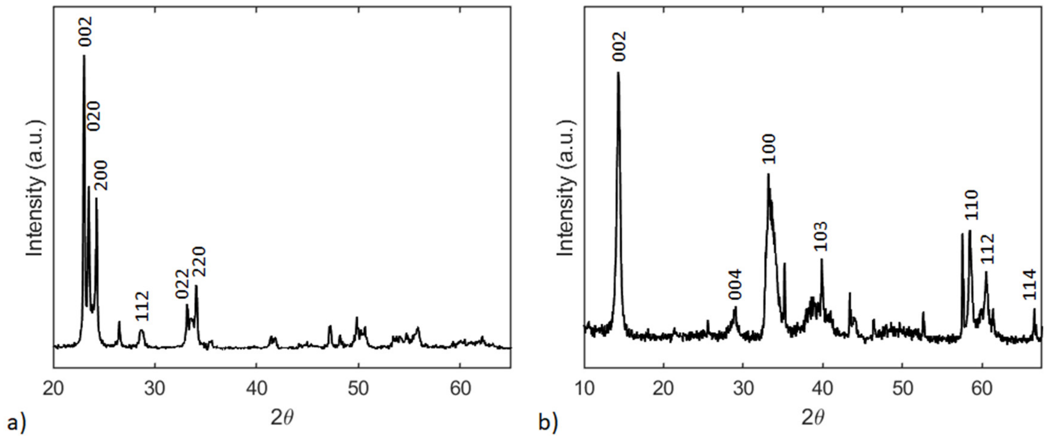

Figure 3. XRD pattern from a) WO_3 pristine sensor and b) WS_2 sensor.

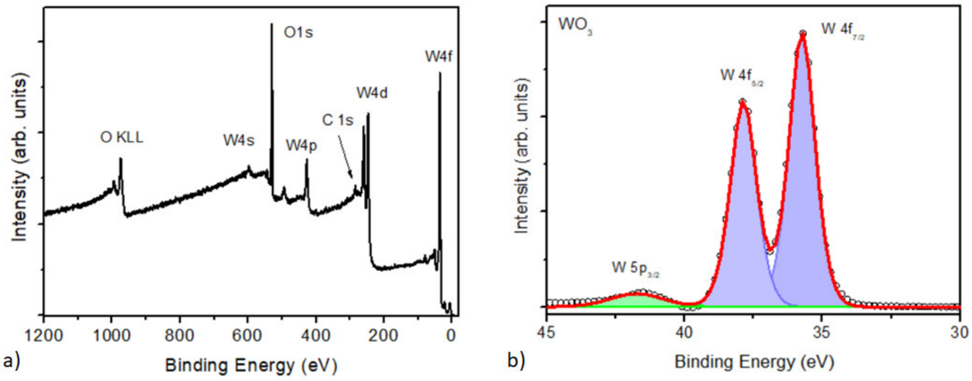

Figure 4. (a) $SrTiO_3$@WO_3 sample XPS survey spectrum. The relative concentration of the identified elements (O 24% at., W 70% at. and C 6% at.). (b) XPS spectrum recorded in the W 4f binding energy region. The doublet with the W $4f_{7/2}$ component centered at 35.5 eV and the W $4f_{5/2}$ at 37.7 eV, is generated by photoelectrons emitted from W atoms with oxidation state + 6 (WO_3).

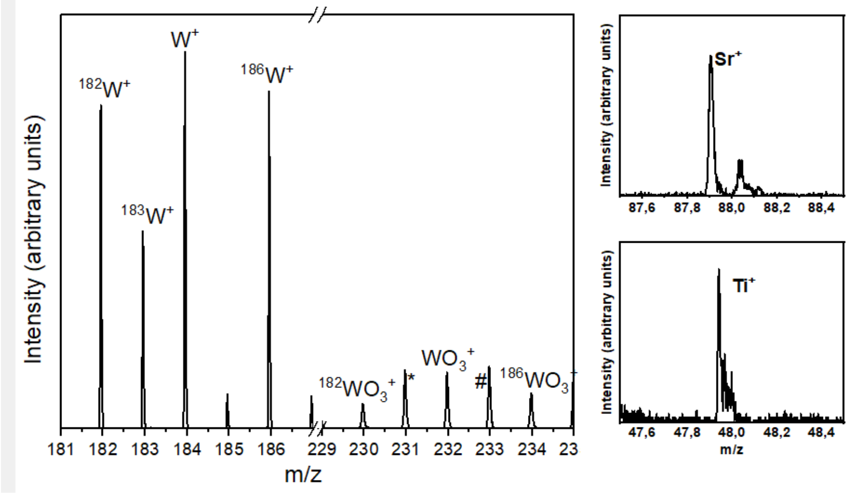

Figure 5. ToF-SIMS analysis spectrum from the $SrTiO_3$@WO_3 sensor.

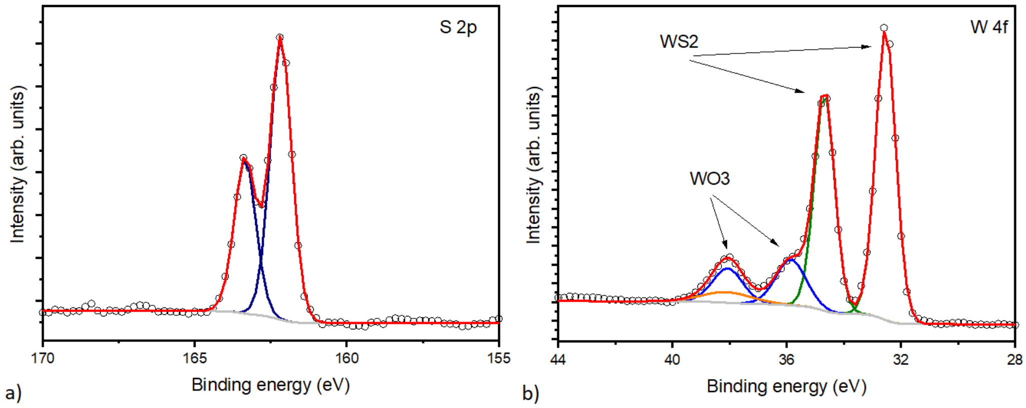

Figure 6. (a) XPS spectrum recorded in the S 2p binding energy region. The peaks related to the S $2p_{1/2}$ and S $2p_{3/2}$ orbital of divalent sulfide ions are observed at 163.3 and 162.1 eV. (b) XPS spectrum recorded in the W 4f binding energy region. The peaks located at 38.3, 34.7, and 32.5 eV correspond to W $5p_{3/2}$, W$4f_{5/2}$, and W $4f_{7/2}$, respective-ly. The energy positions of these peaks indicate a W valence of +4, which is in accordance with the previous reports. The other doublet with components at W$4f_{5/2}$, and W $4f_{7/2}$ respectively at 30.8 and 35.8 eV indicates the presence of W-O in WO_3.

Standard measurements

The different sensors were characterized for gas sensing using the traditional heating activation mechanism. Measurements were done using the same gas sensing system presented in the manuscript. The operating temperatures tested were 50, 100, and 150 °C. The three sensors were exposed at 25, 50, and 75 ppm of NH_3, and 250, 500, and 750 ppb of NO_2 to check the sensing performance towards reducing and oxidizing gases. Figure S7 shows the sensors' response at 150 °C when the WS_2 (p-type) sensor is exposed to NH_3 (reducing gas) and WO_3 and $SrTiO_3$ doped WO_3 (n-type) sensors are exposed to NO_2 (oxidizing gas). Although the WO_3 sensor presents the higher response its standard deviation represents about 30% of the mean value for each concentration. On the other hand, WS_2 and $SrTiO_3$ doped sensors present a lower response but the standard deviation is lower than 2% of the average value for most of the measured concentrations. Measurement at

150 °C present a higher response and lower standard deviation than those when the operating temperature was set at 50 and 150 °C.

Measurements performed using the p-type sensor towards the oxidizing gas and n-type sensors towards the reducing gas presented either almost no response or an unusual behavior where sensor response highest values were obtained for the lower concentrations measured.

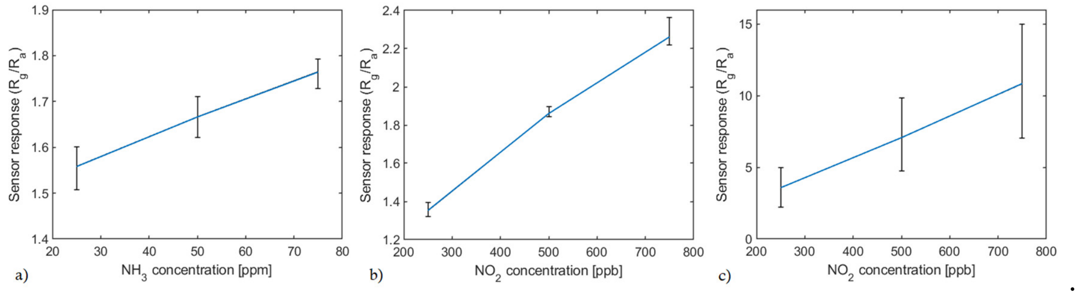

Figure 7. Sensors' response working under standard heating activation at 150 °C. a) WS_2 response for NH_3. b) $SrTiO_3$@WO_3 response for NO_2. c) WO_3 response for NO_2.

Principal component analysis for gas identification

The target gas identification process was performed creating training matrixes including NO_2 and NH_3 observations and using frequency components from the three synthesized sensors. The sensor's performance when these are operated under both, visible and UV light modulation was analyzed. PCA scores and loadings from the PC1 and PC2 were used to create biplots that allow discrimination between the sensors and frequency components which better fit the gas identification. Figures S8a and b depict the PCA scores and loadings biplots from principal components analysis carried out building the training matrix using frequency components related to the three synthesized sensors and observations from NO_2 and NH_3 when sensors work under visible and UV light modulation. Under visible light modulation (Figure S8a), the loadings related to the light switching frequency from the WO_3 pristine sensor and the $SrTiO_3$@WO_3 sensor are well correlated and appear oriented in the direction of the PC1. For its part, loading related to its respective even order harmonics are not likely to be correlated with the switching frequency components, as their loading are orthogonal (appear at 90° across the center). NO_2 and NH_3 observations are separated in clusters according to the PC1 and the NO_2 different concentrations are also separated in clusters according to the same direction. Moreover, loadings from the WS_2 sensor related to the light switching frequency and its even order harmonics are oriented in the PC2 direction and too close to the origin, which means that the information from these components are less relevant in the gas identification. Under UV light modulation (Figure S8b), the loadings from the WS_2 sensor related to all the frequencies are also oriented in the PC2 direction and too close to the origin. On the other hand, loadings related to the switching frequency from the WO_3 and $SrTiO_3$@WO_3 sensors are not likely to be correlated, since these are orthogonal. The two loadings have an important weight on the PC1. Loadings from these two sensors related to the even order harmonics have a positive correlation with their light switching frequencies. In this case, the NO_2 and NH_3 observations are also separated according to the PC1, allowing to clearly identify the gas to which the sensors are exposed. Focusing on the clusters formed by the different NO_2 concentrations, these are oriented perpendicular to the $SrTiO_3$@WO_3 switching frequency loading direction. Following this direction, it is also possible to separate clusters of different NO_2 observations. In order to support and clarify this result, PCA biplots were developed using two different training matrixes. Both matrixes use observations of NO_2 and NH_3, but one includes just frequency components related to the WO_3 sensor (Figure S8 c and d) and the other includes just frequency components related to the $SrTiO_3$@WO_3 sensor (Figure S8e and f). Results obtained show how under UV light modulation, it is possible to separate different NO_2 concentration observations using the

$SrTiO_3@WO_3$ sensor, while using the WO_3 all the observations appear together in one cluster. This makes the $SrTiO_3@WO_3$ more suitable to be used in the gas identification process since it is not only suitable to identify observations from NO_2 and NH_3, but it also allows to separate NO_2 concentrations under both visible and UV light modulation.

On the other hand, with the purpose of focusing just on the NH_3 different concentrations distribution, new training matrixes were built using just observation of this gas. The matrixes were made of four frequency components of the three synthesized sensors. The PCA biplots (shown in Figure S9) for the modulation under both, visible and UV light were evaluated, and frequency component loadings show the same behavior. The loading related to the light switching frequency and its even order harmonics for the WS_2 sensor are well correlated and are oriented in the direction of the PC2. Loadings related to the switching frequency for the $SrTiO_3@WO_3$ and the WO_3 sensors are not likely to be correlated with the one related to the same frequency for the WS_2 sensor, since they meet each other at about 90 °. The loadings related to the even order harmonics from these sensors are located near the origin, so the information they give to the discrimination process is not relevant. In addition, NH_3 different concentration observations appear to be organized according to the PC2. Hence, the classification of the different NH_3 concentrations is related to the frequency components from the WS_2 sensor. Since the loadings related to the even harmonics from the WS_2 sensor are located very close one each other, it is possible to use just the switching frequency and its first even order harmonic to quantify the different NH_3 concentrations.

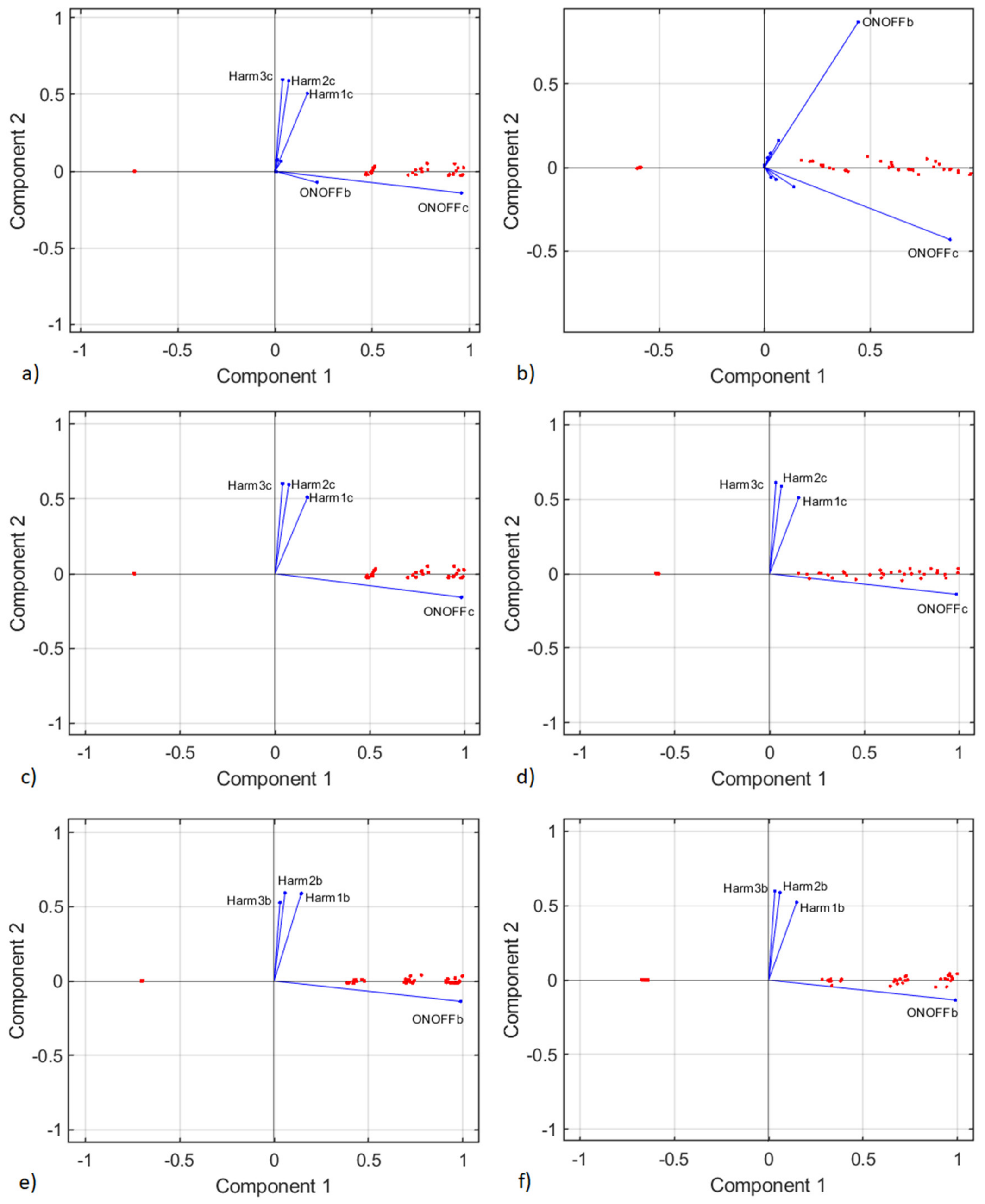

Figure 8. PCA performed with observations related to NO_2 and NH_3. PCA biplots from the left column belong to visible light modulation and those from right column to UV light modulation. Subplots a and b were obtained using a training matrix made of four frequency components from each of the three synthesized sensors. Subplots c and d belong to training matrix built with frequency components from the WO_3 pristine sensor, while e and f use frequency components from the $SrTiO_3@WO_3$ sensor. Loadings identified with the letter 'b' are related to frequency components from the $SrTiO_3$ sensor, while the letter 'c' identifies the loadings related to frequency components from WO_3 sensor.

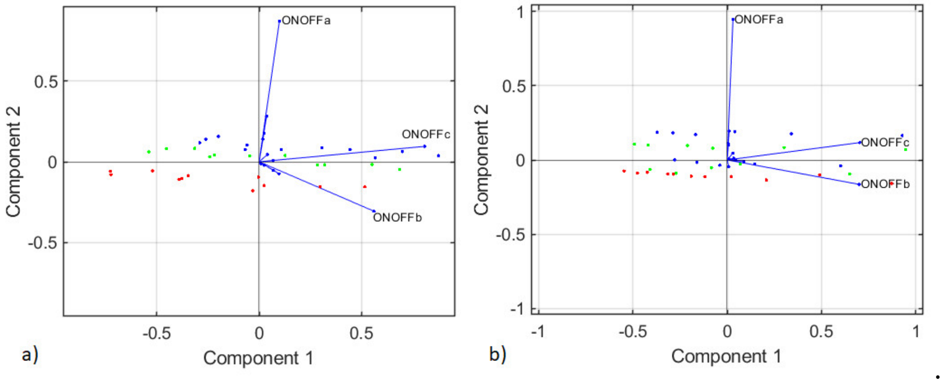

Figure 9. PCA performed using observations of just NH_3 concentrations and four frequency components of each of the three synthesized sensors. Frequency components identified with the letter 'a' are related to the WS_2 sensor, the letter 'b' identifies the frequency components related to the $SrTiO_3@WO_3$ sensor, and letter 'c' the WO_3 pristine sensor. Red markers identify the 25 ppm observations, green markers the 50 ppm observations, and blue markers the 75 ppm observations.

PCR calibration models

The WO_3 sensor PCR model results for NO2 are shown in Figure S10. In this case, when visible light modulation is applied the R2 value is about 0.97, and the RMSE value is about 7% of the total measured concentration range, which makes the model accurate to predict NO_2 gas concentrations. On the other hand, when the light modulation is performed using UV LEDs the model presents an R2 value of 0.84 and its RMSE value represents more than 16 % of the total concentration variation in the set of measurements. This shows the same behavior as the WS_2 and $SrTiO_3@WO_3$ sensors, where the concentration prediction performance is better when sensors work under visible light modulation.

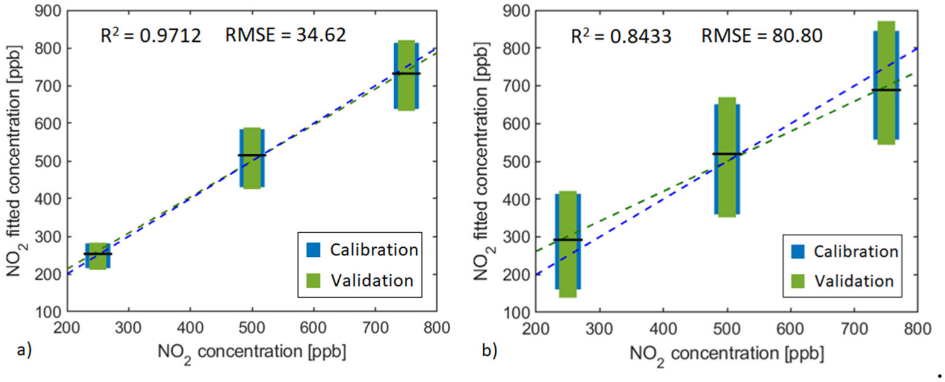

Figure 10. PCR calibration model and cross-validation for the $SrTiO_3$ doped WO_3 sensor towards NO_2 concentrations under a) visible light modulation and b) UV light modulation. Blue boxed represents the calibration model dispersion for each concentration and green boxes the validation dispersion. The horizontal black line represents the mean value for the validation process. The validation linear fit is shown with the green dashed line, and the blue dashed line represents a unitary slope line. R-squared and RMSE values belong to the calibration model.

The efficacy of n-type sensors and p-type sensors to respectively quantify reducing and oxidizing gases was also estimated. In this case, as the previous results obtained, where just temperature was applied to activate the sensing layer, were unsatisfactory to accomplish the goal of the present work, models were performed using the maximum possible number of frequency components and principal components. Thus, to get the best R^2 and RMSE values from the models. From the results presented in Figure S11, it is evident that n-type sensors and p-type sensors are not useful for predicting reducing and oxidizing gases using the methodology presented in this work. Hence, being that also consequent with the results obtained when sensors are operated at just a relatively low temperature without light modulation.

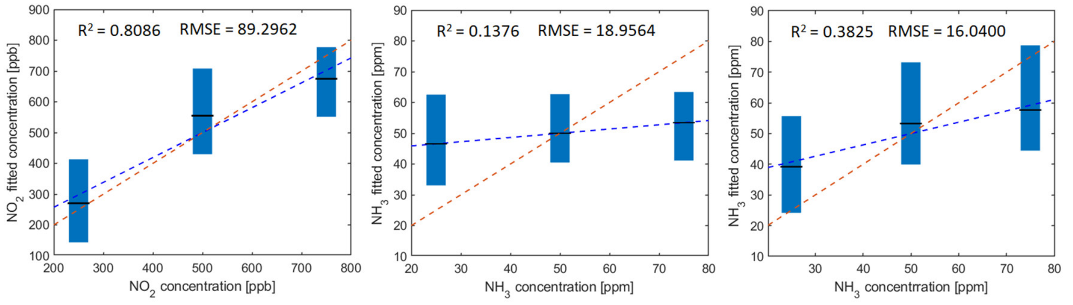

Figure 11. PCR calibration model for the a) WS_2 sensor towards NO_2 concentrations, b) $SrTiO_3$@WO_3 sensor towards NH_3 concentrations and c) WO_3 sensor towards NH_3 concentrations. The operating temperature was 50 °C and the light modulation was done with purple visible light LEDs. Blue boxed represents the calibration model dispersion for each concentration. The horizontal black line represents the mean value for the validation process. The calibration linear fit is shown with the blue dashed line, and the orange dashed line represents a unitary slope line.
